# Supplementary material for: Decreased inflammatory cytokine production of antigen-specific CD4+ T cells in NMDA receptor encephalitis
Source: J Neurol. 2021 Jan 13;268(6):2123–31. doi: 10.1007/s00415-020-10371-y (PMC8179900; doi:10.1007/s00415-020-10371-y)
Supplement: Supplementary file 1 — Supplementary material 1 (DOCX 507 kb) [file 415_2020_10371_MOESM1_ESM.pdf]

Constructs	Sequences	Amino acids
NR1 full-length<br>ATD<br>NR1 commercial<br>S1S2	MSTMRLLTLA LLFSCSVARA ACDPKIVNIG AVLSTRKHEQ MFREAVNQAN KRHGSWKIQL NATSVTHKPN AIQMALSVCE DLISSQVYAI LVSHPPTPND<br>MSTMRLLTLA LLFSCSVARA ACDPKIVNIG AVLSTRKHEQ MFREAVNQAN KRHGSWKIQL NATSVTHKPN AIQMALSVCE DLISSQVYAI LVSHPPTPND<br>---------- --------RA ACDPKIVNIG AVLSTRKHEQ MFREAVNQAN KRHGSWKIQL NATSVTHKPN AIQMALSVCE DLISSQVYAI LVSHPPTPND<br>---------- ---------- ---------- ---------- ---------- ---------- ---------- ---------- ---------- ----------	1-100
	HFTPTPVSYT AGFYRIPVLG LTTRMSIYSD KSIHLSFLRT VPPYSHQSSV WFEMMRVYSW NHIILLVSDD HEGRAAQKRL ETLLEERESK AEKVLQFDPG<br>HFTPTPVSYT AGFYRIPVLG LTTRMSIYSD KSIHLSFLRT VPPYSHQSSV WFEMMRVYSW NHIILLVSDD HEGRAAQKRL ETLLEERESK AEKVLQFDPG<br>HFTPTPVSYT AGFYRIPVLG LTTRMSIYSD KSIHLSFLRT VPPYSHQSSV WFEMMRVYSW NHIILLVSDD HEGRAAQKRL ETLLEERESK AEKVLQFDPG<br>---------- ---------- ---------- ---------- ---------- ---------- ---------- ---------- ---------- ----------	101-200
	TKNVTALLME AKELEARVII LSASEDDAAT VYRAAAMLNM TGSGYVWLVG EREISGNALR YAPDGILGLQ LINGKNESAH ISDAVGVVAQ AVHELLEKEN<br>TKNVTALLME AKELEARVII LSASEDDAAT VYRAAAMLNM TGSGYVWLVG EREISGNALR YAPDGILGLQ LINGKNESAH ISDAVGVVAQ AVHELLEKEN<br>TKNVTALLME AKELEARVII LSASEDDAAT VYRAAAMLNM TGSGYVWLVG EREISGNALR YAPDGILGLQ LINGKNESAH ISDAVGVVAQ AVHELLEKEN<br>---------- ---------- ---------- ---------- ---------- ---------- ---------- ---------- ---------- ----------	201-300
	ITDPPRGCVG NTNIWKTGPL FKRVLMSSKY ADGVTGRVEF NEDGDRKFAN YSIMNLQNRK LVQVGIYNGT HVIPNDRKII WPGGETEKPR GYQMSTRLKI<br>ITDPPRGCVG NTNIWKTGPL FKRVLMSSKY ADGVTGRVEF NEDGDRKFAN YSIMNLQNRK LVQVGIYNGT HVIPNDRKII WPGGETEKPR ----------<br>ITDPPRGCVG NTNIWKTGPL FKRVLMSSKY ADGVTGRVEF NEDGDRKFAN YSIMNLQNRK LVQVGIYNGT HVIPNDRKII WPGGETEKPR GYQMSTRLKI<br>---------- ---------- ---------- ---------- ---------- ---------- ---------- ---------- ---------- GYQMSTRLKI	301-400
	VTIHQEPFVY VKPTLSDGTC KEEFTVNGDP VKKVICTGPN DTSPGSPRHT VPQCCYGFCI DLLIKLARTM NFTYEVHLVA DGKFGTQERV NNSNKKEWNG<br>---------- ---------- ---------- ---------- ---------- ---------- ---------- ---------- ---------- ----------<br>VTIHQEPFVY VKPTLSDGTC KEEFTVNGDP VKKVICTGPN DTSPGSPRHT VPQCCYGFCI DLLIKLARTM NFTYEVHLVA DGKFGTQERV NNSNKKEWNG<br>VTIHQEPFVY VKPTLSDGTC KEEFTVNGDP VKKVICTGPN DTSPGSPRHT VPQCCYGFCI DLLIKLARTM NFTYEVHLVA DGKFGTQERV NNSNKKEWNG	401-500
	MMGELLSGQA DMIVAPLTIN NERAQYIEFS KPFKYQGLTI LVKKEIPRST LDSFMQPFQS TLWLLVGLSV HVVAVMLYLL DRFSPFGRFK VNSEEEEEDA<br>---------- ---------- ---------- ---------- ---------- ---------- ---------- ---------- ---------- ----------<br>MMGELLSGQA DMIVAPLTIN NERAQYIEFS KPFKYQGLTI LVKKEIPRST LDSFMQPFQ- ---------- ---------- ---------- ----------<br>MMGELLSGQA DMIVAPLTIN NERAQYIEFS KPFKYQGLTI LVKK------ ---------- ---------- ---------- ---------- ----------	501-600
	LTLSSAMWFS WGVLLNSGIG EGAPRSFSAR ILGMVWAGFA MIIVASYTAN LAAFLVLDRP EERITGINDP RLRNPSDKFI YATVKQSSVD IYFRRQVELS<br>---------- ---------- ---------- ---------- ---------- ---------- ---------- ---------- ---------- ----------<br>---------- ---------- ---------- ---------- ---------- ---------- ---------- ---------- ---------- ----------<br>---------- ---------- ---------- ---------- ---------- ---------- EERITGINDP RLRNPSDKFI YATVKQSSVD IYFRRQVELS	601-700
	TMYRHMEKHN YESAAEAIQA VRDNKLHAFI WDSAVLEFEA SQKCDLVTTG ELFFRSGFGI GMRKDSPWKQ NVSLSILKSH ENGFMEDLDK TWVRYQECDS<br>---------- ---------- ---------- ---------- ---------- ---------- ---------- ---------- ---------- ----------<br>---------- ---------- ---------- ---------- ---------- ---------- ---------- ---------- ---------- ----------<br>TMYRHMEKHN YESAAEAIQA VRDNKLHAFI WDSAVLEFEA SQKCDLVTTG ELFFRSGFGI GMRKDSPWKQ NVSLSILKSH ENGFMEDLDK TWVRYQECDS	701-800
	RSNAPATLTF ENMAGVFMLV AGGIVAGIFL IFIEIAYKRH KDARRKQMQL AFAAVNVWRK NLQDRKSGRA EPDPKKKATF RAITSTLASS FKRRRSSKDT<br>---------- ---------- ---------- ---------- ---------- ---------- ---------- ---------- ---------- ----------<br>---------- ---------- ---------- ---------- ---------- ---------- ---------- ---------- ---------- ----------	801-900

	---------- ---------- ---------- ---------- ---------- ---------- ---------- ---------- ---------- ----------	
	STGGGRGALQ NQKDTVLPRR AIEREEGQLQ LCSRHRES<br>---------- ---------- ---------- --------<br>---------- ---------- ---------- --------<br>---------- ---------- ---------- --------	901-930
LGI1 commercial	---------- ---------- ---------- ----KKPAKP KCPAVCTCTK DNALCENARS IPRTVPPDVI SLSFVRSGFT EISEGSFLFT PSLQLLLFTS<br>NSFDVISDDA FIGLPHLEYL FIENNNIKSI SRHTFRGLKS LIHLSLANNN LQTLPKDIFK GLDSLTNVDL RGNSFNCDCK LKWLVEWLGH TNATVEDIYC<br>EGPPEYKKRK INSLSSKDFD CIITEFAKSQ DLPYQSLSID TFSYLNDEYV VIAQPFTGKC IFLEWDHVEK TFRNYDNITG TSTVVCKPIV IETQLYVIVA<br>QLFGGSHIYK RDSFANKFIK IQDIEILKIR KPNDIETFKI ENNWYFVVAD SSKAGFTTIY KWNGNGFYSH QSLHAWYRDT DVEYLEIVRT PQTLRTPHLI<br>LSSSSQRPVI YQWNKATQSF TNQTDIPNME DVYAVKHFSV KGDVYICLTR FIGDSKVMKW GGSSFQDIQR MPSRGSMVFQ PLQINNYQYA ILGSDYSFTQ<br>VYNWDAEKAK FVKFQELNVQ APRSFTHVSI NKRNFLFASS FKGNTQIYKH VIVDLSA	1-100<br>101-200<br>201-300<br>301-400<br>401-500<br>501-557

Supplementary Table 1

Protein sequences underlying the antigens used for T cell stimulation. *NR1 full-length*, sequence of the NR1 subunit of the NMDAR protein for comparison; *ATD*, aminoterminal domain of the NR1 subunit; *NR1 commercial*, recombinant NR1 protein (MyBiosource, #964741); *S1S2*, S1 and S2 extracellular part of the NR1 subunit; *LGI1 commercial*, recombinant LGI1 protein (MyBiosource, #1378533).
